# Boosting BCG with Recombinant Modified Vaccinia Ankara Expressing Antigen 85A: Different Boosting Intervals and Implications for Efficacy Trials

**DOI:** 10.1371/journal.pone.0001052

**Published:** 2007-10-24

**Authors:** Ansar A. Pathan, Clare R. Sander, Helen A. Fletcher, Ian Poulton, Nicola C. Alder, Natalie E. R. Beveridge, Kathryn T. Whelan, Adrian V. S. Hill, Helen McShane

**Affiliations:** 1 Centre for Clinical Vaccinology and Tropical Medicine, University of Oxford, Churchill Hospital, Oxford, United Kingdom; 2 Centre for Statistics in Medicine, Wolfson College Annexe, University of Oxford, Oxford, United Kingdom; University of Maryland School of Medicine, United States of America

## Abstract

**Objectives:**

To investigate the safety and immunogenicity of boosting BCG with modified vaccinia Ankara expressing antigen 85A (MVA85A), shortly after BCG vaccination, and to compare this first with the immunogenicity of BCG vaccination alone and second with a previous clinical trial where MVA85A was administered more than 10 years after BCG vaccination.

**Design:**

There are two clinical trials reported here: a Phase I observational trial with MVA85A; and a Phase IV observational trial with BCG. These clinical trials were all conducted in the UK in healthy, HIV negative, BCG naïve adults. Subjects were vaccinated with BCG alone; or BCG and then subsequently boosted with MVA85A four weeks later (short interval). The outcome measures, safety and immunogenicity, were monitored for six months. The immunogenicity results from this short interval BCG prime–MVA85A boost trial were compared first with the BCG alone trial and second with a previous clinical trial where MVA85A vaccination was administered many years after vaccination with BCG.

**Results:**

MVA85A was safe and highly immunogenic when administered to subjects who had recently received BCG vaccination. When the short interval trial data presented here were compared with the previous long interval trial data, there were no significant differences in the magnitude of immune responses generated when MVA85A was administered shortly after, or many years after BCG vaccination.

**Conclusions:**

The clinical trial data presented here provides further evidence of the ability of MVA85A to boost BCG primed immune responses. This boosting potential is not influenced by the time interval between prior BCG vaccination and boosting with MVA85A. These findings have important implications for the design of efficacy trials with MVA85A. Boosting BCG induced anti-mycobacterial immunity in either infancy or adolescence are both potential applications for this vaccine, given the immunological data presented here.

**Trial Registration:**

ClinicalTrials.gov NCT00427453 (short boosting interval), NCT00427830 (long boosting interval), NCT00480714 (BCG alone)

## Introduction

In 2003, there were 8.8 million new cases of tuberculosis (TB) throughout the world and 1.7 million deaths [1]. This makes TB the leading cause of death from a curable disease, despite widespread deployment of the only available vaccine against TB, *Mycobacterium bovis* Bacille Calmette-Guerin (BCG). The protective efficacy of BCG is hugely variable, but overall, BCG fails to protect against pulmonary disease, particularly in adults in the developing world [2]. However, when administered at birth, as it is in most of the developing world, BCG does confer consistent protection against disseminated disease in childhood, and is highly cost effective against severe childhood TB [3], [4]. Ideally any improved vaccine strategy against TB should therefore include BCG. As repeated BCG vaccination does not appear to improve the protective efficacy of a single BCG vaccination, there is an urgent need to develop new improved boosting vaccines [5].

*Mycobacterium tuberculosis* (*M.tb*) is an intracellular pathogen and any new TB vaccine will need to induce high levels of cellular immunity [6]. The secretion of interferon gamma (IFN-γ) from antigen specific T cells is an essential component of the host cellular immune response to *M.tb* and animals and humans with deficiencies in the IFN-γ pathway are profoundly more susceptible to disseminated mycobacterial infections, including *M.tb* [7], [8]. Class II restricted CD4+ T cells are essential for protective immunity, as evident by the increased susceptibility of HIV infected individuals to reactivation of latent *M.tb* infection [9]. Class I restricted CD8+ T cells may also have a role to play [10]. Recombinant viral vectors are highly effective at inducing high levels of both CD4+ and CD8+ T cells [11]–[13]. We have previously shown that MVA85A, the first subunit TB vaccine to enter clinical trials, induces high levels of antigen specific T cells when given alone. We have also shown that a single immunisation with MVA85A is highly effective at boosting BCG induced immune responses in subjects in whom BCG vaccination was administered many years previously[14].

An effective new TB vaccine designed to boost BCG would be used in two ways: either, boosting in infancy (i.e. shortly after BCG vaccination); or, boosting in adolescence (i.e. many years after BCG vaccination). The aim of the first clinical trial presented here was therefore to investigate the safety and immunogenicity of boosting BCG induced immune responses with MVA85A four weeks after BCG vaccination (NCT00427453). We then compared the immunogenicity results from this trial with the second trial presented here, which involves vaccination with BCG alone (NCT00480714). In addition, we then present a discussion comparing the results from this new trial of short interval BCG prime–MVA85A boost (NCT00427453) with the previously published results in which boosting with MVA85A was performed more than 10 years after BCG vaccination (NCT00427830)[14].

## Methods

**Figure 1:**
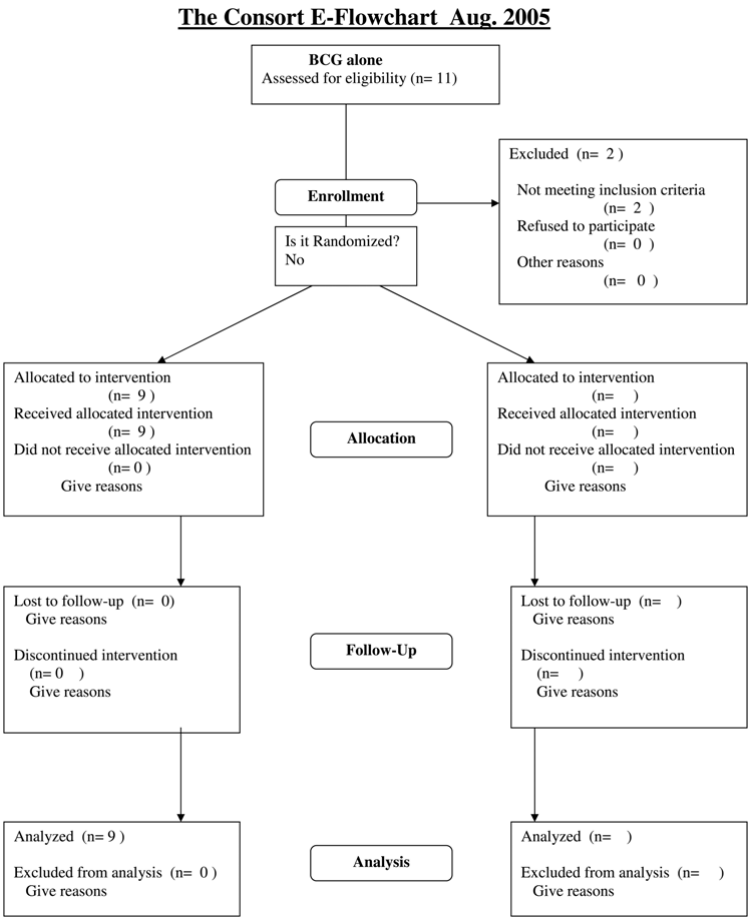
Consort flowchart for the BCG alone trial

The protocols for this trial and supporting CONSORT checklist are available as supporting information; see Checklist S1 and Protocols S1, S2, and S3. Consort flowcharts for each of the three trials discussed here are presented as Figures 1–3.

**Figure 2 pone-0001052-g002:**
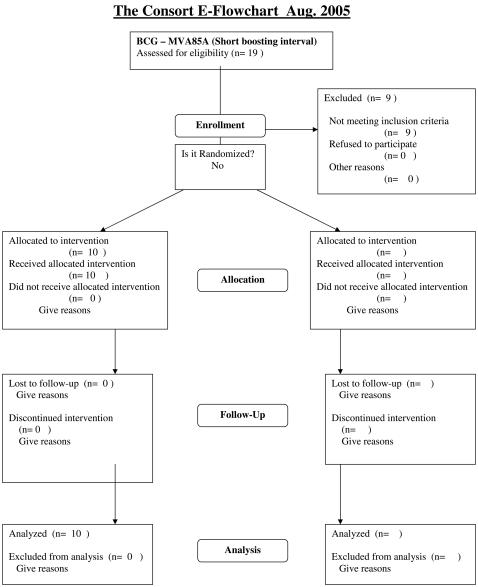
Consort flowchart for the BCG prime–MVA85A boost (short interval) trial

**Figure 3:**
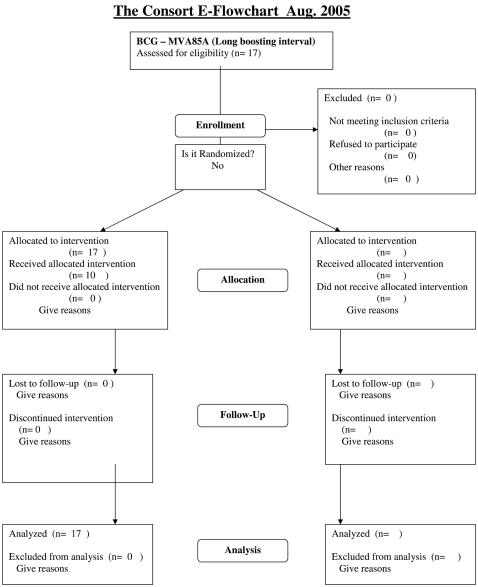
Consort flowchart for the BCG prime–MVA85A boost (long interval) trial

### Participants

Both clinical trials were conducted in BCG naïve; tuberculin skin test negative (Heaf test grade 0 or 1), healthy volunteers. Subjects were recruited for these clinical trials under protocols approved by the Oxfordshire Research Ethics Committee and enrolled only after obtaining written informed consent. They were aged 19–48 (median = 26) and were all seronegative for HIV, HBV and HCV at screening. Demographic information on the subjects is summarised in Table 1. (The data for the middle column (long-boost interval) derive from the trial published in [14]). Routine laboratory haematology and biochemistry were performed prior to vaccination and all values were within normal limits. All subjects enrolled in the trials presented here were negative on an ex-vivo Elispot assay for the two *M.tb* specific antigens ESAT6 and CFP10.

**Table 1:** Demographic details of subjects according to trial. The data for the middle column (long-boost interval) derive from the trial published in [14]

	BCG–MVA85A (short boosting interval) (n = 10)	BCG–MVA85A (long boosting interval) (n = 17)	BCG alone (n = 9)
**Male**	30%	53%	22%
**Age yrs: Mean (SD)**	27 (7.2)	31 (11.4)	27 (5.6)
**Country of birth (%)**			
**UK**	60%	76%	78%
**Africa**	10%	12%	0%
**Europe**	20%	12%	22%
**USA**	10%	0%	0%
**Healthcare worker**	40%	24%	0%
**Significant travel history**	30%	24%	11%

### Interventions

In the first study, volunteers were vaccinated with BCG (a single immunisation with BCG SSI strain, 100 µl administered intra-dermally, *n* = 9). In the second study, volunteers were vaccinated with BCG (SSI strain, 100 µl administered intra-dermally) and 4 weeks later were vaccinated with a single immunisation with 5×10^7^ pfu MVA85A, intradermally into the contralateral arm to the BCG immunisation (n = 10).

The construction of MVA85A has previously been described [15]. Clinical grade MVA85A was produced under good manufacturing practices by Impfstoffwerke Dessau-Tornau. A Doctors and Dentists Exemption Certificate was issued from the Medicines and Healthcare products Regulatory Agency, London, for the use of MVA85A in clinical trials. The trials reported here completed enrolment before the advent of the UK Medicines for Human Use (Clinical Trials Regulations) 2004.

All the trials reported here were single-arm, non-randomized trials.

### Objectives

The primary outcome measure in the short interval BCG–MVA85A trial presented here was safety and the secondary outcome was vaccine induced cellular immune responses, as measured by an ex-vivo IFN-γ ELISpot assay. All subjects who were vaccinated with MVA85A completed a diary card recording local and systemic side effects and body temperature for 7 days following vaccination.

### Immunogenicity measures

The *ex vivo* IFN-γ ELISpot assay was performed on blood taken at the following time points: at screening (prior to the tuberculin skin test), and then at 1, 4, 8 and 24 weeks after vaccination. These measurements were carried out on fresh PBMCs using tuberculin PPD (20 µg/ml, SSI), purified antigen 85 complex (10 µg/ml), and 7 pools of 9–10 15-mer peptides spanning the length of antigen 85A, which overlapped by 10 amino-acids (final concentration of each peptide in the well10 µg/ml.) Briefly, 300,000 PBMCs per well were plated directly onto the ELISpot plate (MAIP, Millipore) in the presence of antigen, and incubated for 18 hours. Streptokinase/Streptodornase and PHA were used in all assays as positive controls and cells and media alone as the negative control. Assays were performed in duplicate and the results were averaged.

#### Analysis of immunogenicity

The ELISpot data were analysed by subtracting the mean number of spots in the medium and cells alone control wells from the mean counts of spots in wells with antigens or peptide pools, and cells. Counts less than 5 spots/well were disregarded. A well was considered positive if the count was at least twice that in the negative control wells and at least 5 spots more than the negative control wells. For the peptide pool wells, the results were summed across all the peptide pools for each volunteer at each time point. This will count twice a T cell that responds to any of the 10-mer overlap regions that occur in two pools with adjacent peptides, as each pool contains non-overlapping peptides. An area under the curve analysis was performed to compare between the two vaccine groups, BCG alone, and BCG prime–MVA85A boost. A Mann-Whitney test was then used for all comparisons between these two groups (1 week and 24 week responses) and a Wilcoxon Signed Ranks Test was used for all comparisons between time points within a group (baseline vs. 1 week, and baseline vs. 24 weeks).

## Results

### Recruitment

Subjects were recruited into the trials reported here from September 2002 to March 2004. Subjects in both the BCG alone and the BCG–MVA85A groups were followed up for safety and immunogenicity for 6 months after vaccination.

### Safety of MVA85A

In total, 10 healthy volunteers were vaccinated with MVA85A, four weeks after receiving vaccination with BCG. Vaccination with MVA85A was well tolerated and there were no serious or severe vaccine related adverse events in any of these trials. As expected from previous trials with this and other recombinant MVAs, all subjects experienced some mild local adverse events [16], [17]. When the safety data from the short interval BCG–MVA85A trial was compared with the previous data from the long interval BCG–MVA85A trial, the frequency of solicited and spontaneously reported local and systemic adverse events was not different between these two groups (Table 2 and 3)[14].

**Table 2:** Local adverse events after MVA85A vaccination

Adverse event	BCG–MVA85A Short boosting interval (n = 10)	BCG–MVA85A Long boosting interval (n = 17)
Redness	10 (100%)	17 (100%)
Pruritus	4 (40%)	10 (59%)
Pain	9 (90%)	17 (100%)
Induration	10 (100%)	17 (100%)

**Table 3:** Systemic adverse events after MVA85A vaccination

Adverse event	BCG–MVA85A Short boosting interval (n = 10)	BCG–MVA85A Long boosting interval (n = 17)
**Fever**	2 (20%)	1 (6%)
**Feverish**	6 (60%)	5 (29%)
**Arthralgia**	2 (20%)	3 (18%)
**Headache**	6 (60%)	6 (35%)
**Myalgia**	8 (80%)	5 (29%)
**Nausea**	3 (30%)	0 (0%)
**Diarrhoea**	3 (30%)	0 (0%)
**Vasovagal syncope**	0 (0%)	0 (0%)
**Axillary LN**	1 (10%)	1 (6%)
**Alterations in haem/biochem**	0 (0%)	0 (0%)

### Cellular immune responses induced by the different vaccination regimens:

#### (a) Short interval BCG-MVA85A

**Figure 4:**
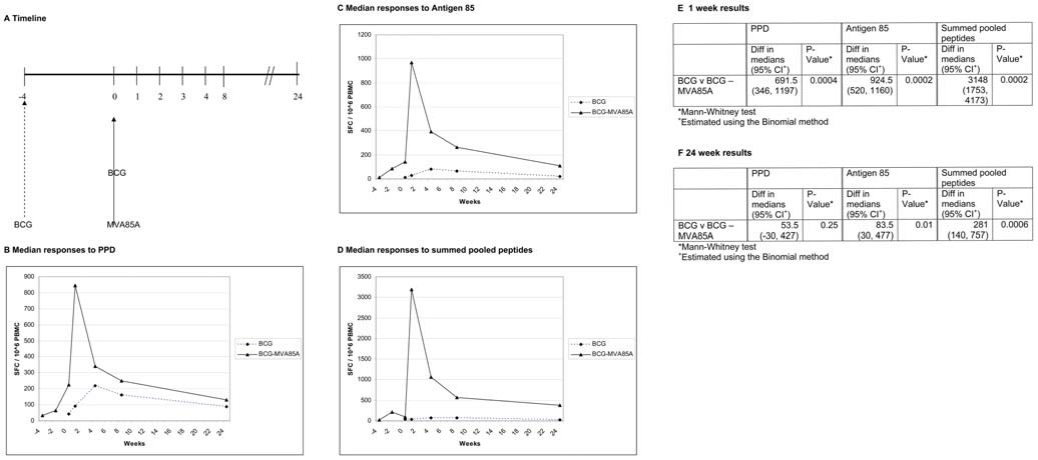
Median IFN-γ ELISpot responses after vaccination in each vaccination group: BCG alone;BCG prime-MVA85A boost. (a) timeline for vaccinations (weeks) in each group; (b)Tuberculin PPD responses; (c) Purified antigen 85 protein responses (d) summed pooled peptide responses; (e) For each of the three antigens measured, the responses between each vaccine group were compared 1 week after vaccination using Mann-Whitney statistic. (f) For each of the three antigens measured, the responses between each vaccine group were compared 24 weeks after vaccination using Mann-Whitney statistic.

When subjects who had been vaccinated 4 weeks previously with BCG were vaccinated with MVA85A, a highly significant rise in antigen specific T cells was seen, 1 week after MVA85A vaccination (PPD, antigen 85 and summed peptide pools, p = 0.005). These responses were all significantly higher in the BCG–MVA85A group than after BCG vaccination alone (Table 4 and Figure 4 b–e). The responses to purified antigen 85 and the summed pooled peptides remained significantly higher than after BCG alone 24 weeks after vaccination (Table 4 and Figure 4 c–d,f). The area under the curve analysis which was performed between day of MVA85A vaccination and 24 weeks showed a significant difference between BCG and BCG-MVA85A for antigen 85 and the summed pooled peptide responses (Tables 5 and 6).

**Table 4:** Median (and inter quartile range) ELISpot responses to PPD, antigen 85 and summed pooled peptides in each vaccination group at each timepoint.

Time after vaccination (weeks)	Median PPD (inter quartile range)		Median Antigen 85 (inter quartile range)		Median Summed pooled peptides (inter quartile range)	
	BCG	BCG-MVA85A	BCG	BCG-MVA85A	BCG	BCG-MVA85A
−4		25 (0 , 124)		12 (2, 33)		35 (8 , 91
−2		219 (19 , 430)		84 (12 , 141)		65 (23 , 221)
0	43 (29 , 155)	224 (53 , 543)	13 (5 , 70)	143 (37 , 274)	32 (30 , 94)	93 (14 , 260)
1	90 (33 , 199)	847 (466 , 1274)	30 (14 , 77)	968 (553 , 1199)	40 (23 , 124)	3189 (1809 , 4253)
4	220 (95 , 354)	340 (175 , 804)	83 (34 , 252)	392 (235 , 922)	67 (45 , 108)	1065 (510 , 1792)
8	160 (127 , 327)	248 (142 , 520)	63 (35 , 84)	264 (131 , 538)	76 (47 , 167)	569 (257 , 1352)
24	87 (50 , 143)	130 (91 , 514)	20 (7 , 84)	110 (68 , 530)	23 (18 , 84)	381 (183 , 798)

**Table 5:** Area under the curve analysis for BCG alone and BCG–MVA85A groups

Vaccine group	n	Median AUC (25^th^ , 75^th ^percentiles)		
		PPD	Antigen 85	Summed pooled peptides
BCG	9	3574 (2599, 7125)	1293 (912, 3201)	1250 (900, 4125)
BCG-MVA85A	10	7289 (3878, 22197)	6900 (4263, 18308)	18500 (10965, 49927)

The area under the curve analysis was carried out between 0 and 24 weeks.

**Table 6:** Comparison of area under the curve analysis for BCG alone and BCG–MVA85A

	PPD		Antigen 85		Summed pooled peptides	
	Diff in medians (95% CI+)	P-Value*	Diff in medians (95% CI+)	P-Value*	Diff in medians (95% CI+)	P-Value*
BCG-MVA85A v BCG	2890 (-130, 10468)	0.06	5023 (2467, 12417)	0.0008	16114 (9671, 27669)	0.0002

* Mann-Whitney test

+ Estimated using the Binomial method

Responses to all these antigenic stimuli were maintained at levels significantly higher than the baseline screening responses, (-4 weeks Fig 4) in the BCG–MVA85A group at 24 weeks after vaccination with MVA85A (PPD p = 0.017; Antigen 85 p = 0.0059; summed peptide pools p = 0.007).

#### (b) Safety and immunogenicity of BCG vaccination

Immunisation with BCG alone in this study induced moderate levels of PPD and antigen 85-specific IFN-γ secreting T cells, which peaked 4 weeks after immunisation (Table 4 and Figure 4b–d). However, the responses to the pooled antigen 85A peptides following BCG vaccination were strikingly weak (Table 4; Figure 4d). At 24 weeks after vaccination, the immune responses induced after BCG vaccination were not significantly different from baseline screening responses (PPD p = 0.110; antigen 85 p = 0.260; summed peptide pools p = 0.236).

The local and systemic adverse event profile after BCG was entirely as expected: all subjects developed a local reaction at the injection site, and there were no systemic adverse reactions.

### Comparison of short interval BCG-MVA85A with long interval BCG-MVA85A

**Figure 5:**
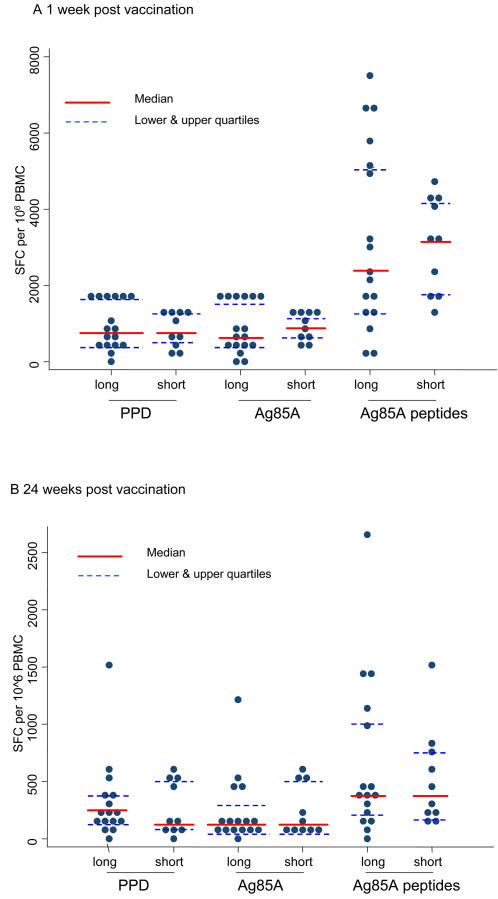
Dot-plot showing data at (a) 1 week and (b) 24 weeks after vaccination in the MVA85A boosted group after recent and distant BCG vaccination. Interval between BCG and MVA85A was four weeks for the short interval trial and a median of 18 years (range 6 months–38 years) in the long interval trial.

The data presented here investigating the immunogenicity of a short boosting interval between BCG vaccination and MVA85A boosting were then compared with the data from the long boosting interval clinical trial previously published [14]. The peak vaccine induced immune response, measured one week after vaccination, was not significantly different between these two clinical trials (PPD p = 0.725; antigen 85 p = 0.92; summed peptide pools p = 0.841, Figure 5(a)). Furthermore, 24 weeks after vaccination, the plateau immune responses were also not significantly different between these two clinical trials (PPD p = 0.635; antigen 85 p = 0.958; summed peptide pools p = 0.937, Figure 5(b)).

## Discussion

There are three important findings from the clinical trials reported here. First, we replicate our previous findings that vaccination with MVA85A in subjects previously vaccinated with BCG is safe and well tolerated. Second, we also replicate our previous findings that BCG induced immune responses can be significantly boosted with MVA85A, as measured by the peak and plateau vaccine induced effector immune responses after vaccination. Third, we show that the boosting potential of MVA85A does not seem to be dependant on interval between BCG vaccination and boosting with MVA85A. We investigated the relationship between boosting interval and peak vaccine induced immune response, 1 week after vaccination. No correlation was found between boosting interval and peak response (Spearman's correlation r = −0.136; p = 0.49, data not shown).

Importantly, these higher antigen specific responses are maintained at a significantly higher level than in the BCG alone arm for at least 24 weeks after vaccination. The same result was found in the previous clinical trial in which a longer boosting interval was investigated. There were no significant differences in plateau responses 24 weeks after vaccination, when the short and long boosting intervals were compared. This persistence of ex-vivo responses cannot be attributed to persistence of the MVA85A vaccine, as MVA85A does not replicate in mammalian cells and does not persist. It is more likely that the MVA85A boost has expanded the memory T cell population, which is either persisting without antigenic exposure or is being constantly re-stimulated or ‘boosted’ by exposure to environmental mycobacteria. We and others have previously demonstrated the existence of anti-mycobacterial immunity induced by environmental mycobacteria in BCG naïve adolescents and adults in the UK [14], [18]. In contrast, the BCG induced immune responses at 24 weeks after vaccination are not significantly different from baseline, despite the fact that BCG will almost certainly persist for longer than MVA85A.

The results of the BCG alone trial are very comparable with our previous data on the immune response induced after BCG vaccination, using the BCG Glaxo strain [14]. Other groups have also found no significant differences between the two strains of BCG used in the UK over the last 5–10 years [19]. Subjects in the short and long boosting interval groups differ in which strain of BCG they were vaccinated with. The short boosting interval subjects presented here were all vaccinated with the SSI strain of BCG, whereas the long boosting interval group previously published were vaccinated with Glaxo BCG. However the comparability of immunogenicity of these two strains found both by us and others suggests that this is not an important factor when comparing the boosting potential of MVA85A between these two groups.

A limitation of the work presented here is that the short boosting interval BCG prime–MVA85A boost trial and the long boosting interval trial previously published were not performed as a single study. In comparing results between trials, the power to detect any differences may be small, particularly given the small sample sizes used in these Phase I studies. However we believe the promising immunogenicity in the short boosting interval trial presented here justifies the further evaluation of this vaccine in efficacy trials as discussed below.

In the absence of a pre-defined immunological correlate of protection, the key question when developing a new TB vaccine is whether such significantly enhanced immune responses seen after the MVA85A boost in both the short and long interval boosting studies are accompanied by an improvement in protective efficacy. This question can only be addressed in large scale efficacy trials. Such trials will need to be conducted in a high incidence population, to obtain efficacy data within a realistic time frame. Even so, these trials are likely to require approximately 10000 subjects and will also require follow-up periods of up to 2 years. A key question when considering the deployment of a new TB vaccine designed to boost BCG is when to administer the boost. One option is to boost in infancy at about 4–6 months of age, and ideally this boost would coincide with an existing EPI schedule vaccine visit, providing no interference occurred between new and existing vaccines. Another potentially useful time to boost BCG is in early adolescence, just before the rise in incidence of TB disease that occurs in adolescence and young adults.

There are two possible efficacy trials which correspond to potential deployment in either infancy or adolescence. Both scenarios have advantages and disadvantages. Boosting in infancy is attractive as there is a well established infrastructure within the EPI for such an additional vaccine. If such a boost were scheduled to coincide with an existing EPI schedule visit, then vaccine take-up would likely be higher. However the major disadvantage with conducting an efficacy trial in infancy (but not necessarily with deployment in this age group once efficacy had been established) is that disease end points can be difficult to define [20]. In contrast, boosting in adolescence is an attractive option as disease endpoints are clearly defined and easy to diagnose in this age group. If effective, boosting in adolescence would have a more immediate impact on the mortality and morbidity of this disease than boosting in infancy. A considerable disadvantage of boosting in adolescence is that there is currently no infrastructure for routine vaccination in this age group, particularly in the developing world. However with the recent licensing of a vaccine against human papilloma virus, scheduled to be administered from 9–15 years of age, such an infrastructure is likely to become established in the future [21]. Ultimately a prophylactic vaccine against HIV would also be likely to be administered in early adolescence.

The aim of these comparative Phase I studies was to investigate the effects of boosting BCG soon after vaccination (thus modelling the infant boosting scenario) and boosting many years after BCG vaccination (thus modelling the adolescent scenario).The immunogenicity data presented here suggest that, at least using this immunological readout, both options of boosting in infancy or of boosting in adolescence may be effective. The data presented here supports the further evaluation of this promising candidate vaccine, which is currently in Phase II clinical trials in South Africa.

## Supporting Information

Protocol S1 BCG alone
(0.12 MB PDF)

Protocol S2 Short interval BCG-MVA85A
(0.12 MB PDF)

Protocol S3 Long boosting BCG-MVA85A
(0.12 MB PDF)

Checklist S1 CONSORT Checklist
(0.05 MB DOC)
