# Correction: Boosting BCG with Recombinant Modified Vaccinia Ankara Expressing Antigen 85A: Different Boosting Intervals and Implications for Efficacy Trials

**DOI:** 10.1371/annotation/71208a01-eb32-469c-96ab-8d5e3c497fce

**Published:** 2011-02-26

**Authors:** Ansar A. Pathan, Clare R. Sander, Helen A. Fletcher, Ian Poulton, Nicola C. Alder, Natalie E. R. Beveridge, Kathryn T. Whelan, Adrian V. S. Hill, Helen McShane

The following information was missing from the Funding section: "The study was supported by funding from the NIHR Oxford Biomedical Research Centre programme."

