# Supplementary material for: Boosting BCG with Recombinant Modified Vaccinia Ankara Expressing Antigen 85A: Different Boosting Intervals and Implications for Efficacy Trials
Source: PLoS One. 2007 Oct 24;2(10):e1052. doi: 10.1371/journal.pone.0001052 (PMC2034536; doi:10.1371/journal.pone.0001052)
Supplement: Protocol S2 — Short interval BCG-MVA85A (0.12 MB PDF) [file pone.0001052.s002.pdf]

University of Oxford

Clinical Trial Protocol

Study reference: 004

A Phase I study of the safety and immunogenicity of a recombinant MVA vaccine encoding a secreted antigen from *M. tuberculosis,* Antigen 85A, delivered intradermally by a needle injection in healthy volunteers who have received BCG immunisation 1 month previously.

CONFIDENTIAL

1. **INTRODUCTION AND BACKGROUND**

1.1 The need for new vaccine against tuberculosis

Tuberculosis (TB) kills about three million people annually. It is estimated that one third of the world's population are latently infected with *Mycobacterium tuberculosis* (*M.tb*). Multi-drug resistant strains of *M.tb*, and co-infection with *M.tb* and HIV present major new challenges. The currently available vaccine, *M. bovis* BCG, is largely ineffective at protecting against adult pulmonary disease in endemic areas and it is widely agreed that a new more effective tuberculosis vaccine is a major global public health priority[1]. However, it may be unethical and impractical to test and deploy a vaccine strategy that does not include BCG, as BCG does confer worthwhile protection against TB meningitis and leprosy. An immunisation strategy that includes BCG is also attractive because the populations in which this vaccine candidate will need to be tested will already have been immunised with BCG.

M.tb is an intracellular organism. CD4+ Th1-type cellular responses are essential for protection and there is increasing evidence from animal and human studies that CD8+ T cells also play a protective role[2]. However, it has generally been difficult to induce strong cellular immune responses in humans using subunit vaccines. DNA vaccines induce both CD4+ and CD8+ T cells and thus offer a potential new approach to a TB vaccine. DNA vaccines encoding various antigens from M. tuberculosis have been evaluated in the murine model, and to date no DNA vaccine alone has been shown to be superior to BCG[3,4].

A heterologous prime-boost immunisation strategy involves giving two different vaccines, each encoding the same antigen, several weeks apart. Such regimes are extremely effective at inducing a cellular immune response. Using a DNA- prime/MVA-boost immunisation strategy induces high levels of CD8+ T cells in animal models of malaria and HIV[5,6], and high levels of both CD4+ and CD8+ T cells in animal models of TB[7]. BCG immunisation alone induces only CD4+ T cells in mice. A prime-boost strategy using BCG as the prime and a recombinant MVA encoding an antigen from *M.tb* that is also present in BCG (antigen 85A: 'MVA85A') as the boost, induces much higher levels of CD4+ T cells than BCG or MVA85A alone. In addition, this regime generates specific CD8+ T cells that are undetectable following immunisation with BCG alone.

1.2 Recombinant viruses as vaccines.

Recombinant viruses used alone have for some years represented a promising vaccine delivery system, particularly for inducing cellular immune responses[8]. The recombinant virus encodes the immunising protein or peptide. Immunisation by a recombinant virus vaccine occurs when host cells take up and express the inoculated attenuated virus encoding a protective antigen[9]. The expressed protein often has the native conformation, glycosylation, and other post-translational modifications that occur during natural infection. Recombinant viral vaccines may elicit both antibody and cytotoxic T-lymphocyte responses[10], which persist without further immunisations. Many viruses have been investigated as potential recombinant vaccines. The successful worldwide eradication of smallpox via vaccination with live vaccinia virus highlighted vaccinia as a candidate for recombinant use[11,12,13]. The recognition in recent years that non-replicating strains of poxvirus such as MVA and avipox vectors can be more immunogenic than traditional replicating vaccinia strains has enhanced the attractiveness of this approach. MVA (modified

vaccinia virus Ankara) is a strain of vaccinia virus which has been passaged more than 570 times though avian cells, is replication incompetent in human cell lines and has a good safety record. It has been administered to more than 120,000 vaccinees as part of the smallpox eradication programme, with no adverse effects, despite the deliberate vaccination of high risk groups[14,15] . This safety in man is consistent with the avirulence of MVA in animal models[16]. MVA has six major genomic deletions compared to the parental genome severely compromising its ability to replicate in mammalian cells[17]. Viral replication is blocked late during infection of cells but importantly viral and recombinant protein synthesis is unimpaired even during this abortive infection[18]. Replication-deficient recombinant MVA has been seen as an exceptionally safe viral vector[19,20]. When tested in animal model studies recombinant MVAs have been shown to be avirulent, yet protectively immunogenic as vaccines against viral diseases and cancer[6,21,22,23,24]. The most useful data on the safety and efficacy of various doses of a recombinant MVA vaccine comes from clinical trial data with a recombinant MVA expressing a number of CTL epitopes from *Plasmodium falciparum* pre-erythrocytic antigens fused to a complete pre-erythrocytic stage antigen, Thrombospondin Related Adhesion Protein (TRAP). These trials have given a total of 169 immunisations with this recombinant MVA, to 49 UK vaccinees 38 Gambian vaccines (20 of whom were children aged 1-5). 6 doses of 1×10^7 pfu, 139 doses of 5×10^7 pfu, 6 doses of 1×10^8 pfu and 18 doses of 2.5×10^8 pfu have been administered, all without serious adverse effects.

1.3 Recombinant MVA encoding antigen 85A

Secreted antigens from *M. tuberculosis* are released from actively metabolising bacteria, and are important targets in protective immunity[25]. Antigen 85A is a major secreted antigen from *M. tuberculosis* which forms part of the antigen 85 complex (A, B and C). This complex constitutes a major portion of the secreted proteins of both *M.tb* and BCG. It is involved in fibronectin binding within the cell wall and has mycolyltransferase activity[26].
MVA85A induces both a CD4+ and a CD8+ epitope when used to immunise mice. When mice are primed with BCG and then given MVA85A as a boost, the levels of CD4+ and CD8+ T cells induced are higher than with either BCG or MVA85A alone.

2. DESCRIPTION OF THE STUDY

2.1 **Study Objective**

To assess the safety and immunogenicity of MVA85A delivered intradermally into the deltoid region in volunteers who have received BCG 1 month previously.

2.2 Selection of volunteers

Volunteers for the study will be recruited through advertisements. Each volunteer will have received an information sheet concerning the study and will have agreed to participate in writing. Volunteers will be given at least 48 hours between reading the information leaflet and agreeing to participate. Female volunteers will be told of the theoretical risk of congenital anomaly should they become pregnant during the study and only those who undertake to take precautions to

avoid pregnancy during the study period will be eligible. Volunteers will give signed consent for their GP's to be notified about their participation in the trial. The GP will be faxed a letter on the day of screening and asked to reply if they know of a reason why the volunteer should not take part. The signed consent form will also be faxed with the letter.

2.3 Screening

Volunteers will be asked to sign the informed consent form for screening. The following will be performed:

- Medical history and examination
- Laboratory evaluations – including clinical chemistry, haematology, HLA typing, anti-vaccinia antibodies, anti-HBV antibodies, anti-HCV antibodies, anti-HIV antibodies
- Heaf test – to exclude prior exposure to TB
- Urinalysis and urine pregnancy test if female

2.4 Inclusion Criteria

- Healthy adult aged 18-55 years.
- Normal medical history and physical examination.
- Normal urine dipstick, blood count, liver enzymes, and creatinine.

2.5 Exclusion Criteria

a. Exposure to TB/BCG vaccination at any point. Previous residence in a TB endemic area.
b. Clinically significant history of skin disorder (eczema, psoriasis, etc.), allergy, immunodeficiency, cardiovascular disease, respiratory disease, endocrine disorder, liver disease, renal disease, gastrointestinal disease, neurological illness, psychiatric disorder, drug or alcohol abuse.
c. Oral or systemic steroid medication or the use of immunosuppressive agents.
d. Positive HIV antibody test, HCV antibody test or positive HBV serology except post-vaccination.
e. Heaf test greater than Grade 0
f. Confirmed pregnancy
g. Previous MVA immunisations

2.6 Withdrawal Criteria

a. Withdrawal of consent by subject for any reason
b. Loss to follow-up
c. Non-compliance with study procedures
d. Protocol violation
e. Serious adverse event (as defined in Appendix 3)
f. Any other reason at discretion of the Principal Investigator
g. Confirmed pregnancy during study period

2.7 Immunisation

On Day 0, subjects will receive a single intradermal injection of 0.1ml BCG (SSI strain) over the deltoid muscle. Blood will be taken at 2 weeks and 4 weeks after this immunisation. At 4 weeks, after blood has been taken, volunteers will be immunised with 5 x 10^7pfu MVA85A in 0.1ml. Subjects will be observed for an hour after MVA85A immunisation. Vital signs will be monitored at 30 and 60 minutes post-immunisation. Local reactions at the site of administration will be evaluated at 60 minutes.

A photograph of the injection site may be taken at 48 hours (with written consent). The injection site will be reviewed 7 days after each immunization.

Blood will be taken at the following time points: At the screening visit*, prior to the BCG vaccination, 2 weeks and 4 weeks after BCG, *1 week after the MVA85A vaccination, 2 weeks, 4 weeks, *8 weeks, and *24 weeks after the vaccination. Up to 55 mls will be taken at any one time with the total being no more than 500 mls over the study period. *Samples taken on these dates will be tested for full blood count and biochemical screen. Immunological assays will be performed at all time points to determine vaccine immunogenicity. A pregnancy test will be performed prior to vaccination for female volunteers. Peripheral blood mononuclear cells will be prepared for cellular immunological assays to be performed without or following cryopreservation. Other serological measures of immune response, i.e. antibody titres, will be assayed on frozen plasma samples.
All blood tests will be taken within 1-3 days of the due date as described in the schedule above.

2.8 Endpoints

The occurance and severity of local side-effects
The occurance and severity of systemic side-effects
The induction of T cell responses (as measured by an interferon-gamma Elispot assay).
Proliferation assays and cytotoxic T cell assays will be performed on strong CD4+ and CD8+ responses respectively.

2.9 Adverse Events

See Appendix 1.

References

1.Colditz GA, Brewer TF, Berkey CS, Wilson ME, Burdick E, Fineberg HV, Mosteller F. Efficacy of BCG vaccine in the prevention of TB. Meta-analysis of the published literature. J Am Med Assoc 1994;271:698-702

2.Stenger S, Modlin RL. T cell mediated immunity to Mycobacterium TB. Curr Op Micro 1999; 2: 89-93

3.Huygen K, Content J, Denis O, Montgomery DL, Yawman AM, Deck RR, DeWitt CM, Orme IM, Baldwin S, D'Souza C, Drowart A, Lozes E, Vandenbussche P, Van Vooren JP, Liu MA, Ulmer JB. Immunogenicity and protective efficacy of a TB DNA vaccine. Nature Medicine 1996;2:893-898

4.Tascon RE, Colston MJ, Ragno S, Stavropoulos E, Gregory D, Lowrie DB. Vaccination against tuberculosis by DNA injection. Nature Medicine 1996;2:888-892

5.Schneider J, Gilbert SC, Blanchard TJ, Hanke T, Robson KJ, Hannan CM, Becker M, Sinden R, Smith GL, Hill AVS. Enhanced immunogenicity for CD8+ T cell induction and complete protective efficacy of malaria DNA vaccination by boosting with modified vaccinia virus Ankara. Nature Medicine 1998;4:397-402

6.Hanke T, Samuel RV, Blanchard TJ, Neumann VC, Allen TM, Boyson JE, Sharpe SA, Cook N, Smith GL, Watkins DI, Cranage MP, McMichael AJ. Effective induction of simian immunodeficiency virus-specific cytotoxic T lymphocytes in macaques by using a multiepitope gene and DNA prime-modified vaccinia virus Ankara boost vaccination regimen. J Virol 1999;73(9):7524-32

7.McShane H, Brookes R, Gilbert SC, Hill AVS. Enhanced immunogenicity of CD4+ T cell responses and protective efficacy of a DNA-MVA prime-boost vaccination regime in murine tuberculosis. Infect Immun submitted

8.Paoletti E. Applications of pox virus vectors to vaccination: an update. Proc Natl Acad Sci USA 1996;93:11349-11353.

9.Smith GL, Cheng KC, Moss B. Vaccinia virus: an expression vector for genes from parasites. Parasitology 1986;92 Suppl:S109-17

10.Rodrigues M, Li S, Murata K, Rodriguez D, Rodriguez JR, Bacik I, Bennink JR, Yewdell JW, Garcia-Sastre A, Nussenzweig RS, et al. Influenza and vaccinia viruses expressing malaria CD8+ T and B cell epitopes. Comparison of their immunogenicity and capacity to induce protective immunity. J Immunol 1994 Nov 15;153(10):4636-48

11.Mackett M, Smith G, Moss B. Vaccinia virus: a selectable eukaryotic cloning and expression vector. Proc Natl Acad Sci USA 1982; 79: 7415-7419.

12.Panicali D, Paoletti E. Construction of poxviruses as cloning vectors: insertion of the thymidine kinase gene from herpes simplex virus into the DNA of infectious vaccinia virus. Proc Natl Acad Sci USA 1982; 79: 4927-4931.

13. Moss B. Genetically engineered poxviruses for recombinant gene expression, vaccination and safety. Proc Natl Acad Sci USA 1996; 93: 11341-11348.

14.Stickl H, Hochstein-Mintzel V, Mayr A, Huber HC, Schafer H, Holzner A. MVA vaccination against smallpox: clinical tests with an attenuated live vaccinia virus strain. Dtsch Med Wochenschr 1974 Nov 22;99(47):2386-92.

15.Mahnel H, Mayr A. Experiences with immunization against orthopox viruses of humans and animals using vaccine strain MVA. Berl Munch Tierarztl Wochenschr 1994 Aug;107(8):253-6.

16.Mayr A, Stickl H, Muller HK, Danner K, Singer H. The smallpox vaccination strain MVA: marker, genetic structure, experience gained with the parenteral vaccination and behavior in organisms with a debilitated defence mechanism. Zentralbl Bakteriol [B] 1978 Dec;167(5-6):375-90.
17.Meyer H, Sutter G, Mayr A. Mapping of deletions in the genome of the highly attenuated vaccinia virus MVA and their influence on virulence. J Gen Virol 1991 May;72 (Pt 5):1031-8.
18.Sutter G, Moss B. Nonreplicating vaccinia vector efficiently expresses recombinant genes. Proc Natl Acad Sci U S A 1992 Nov 15;89(22):10847-51.
19. Moss B. Genetically engineered poxviruses for recombinant gene expression, vaccination and safety. Proc Natl Acad Sci USA 1996; 93: 11341-11348.
20. Sutter G, Moss B. Novel vaccinia vector derived from the host range restricted and highly attenuated MVA strain of vaccinia virus. Dev Biol Stand 1995;84:195-200.
21.Sutter G, Wyatt LS, Foley PL, Bennink JR, Moss B. A recombinant vector derived from the host range-restricted and highly attenuated MVA strain of vaccinia virus stimulates protective immunity in mice to influenza virus. Vaccine 1994 Aug;12(11):1032-40.
22.Hirsch VM, Fuerst TR, Sutter G, Carroll MW, Yang LC, Goldstein S, Piatak M Jr, Elkins WR, Alvord WG, Montefiori DC, Moss B, Lifson JD. Patterns of viral replication correlate with outcome in simian immunodeficiency virus (SIV)-infected macaques: effect of prior immunization with a trivalent SIV vaccine in modified vaccinia virus Ankara. J Virol 1996 Jun;70(6):3741-52.
23.Wyatt LS, Shors ST, Murphy BR, Moss B. Development of a replication-deficient recombinant vaccinia virus vaccine effective against parainfluenza virus 3 infection in an animal model. Vaccine 1996 Oct;14(15):1451-8.
24.Carroll MW, Overwijk WW, Chamberlain RS, Rosenberg SA, Moss B, Restifo NP. Highly attenuated modified vaccinia virus Ankara (MVA) as an effective recombinant vector: a murine tumor model. Vaccine 1997 Mar;15(4):387-94.
25. Horwitz MA, Lee BW, Dillon BJ, Harth G. Protective immunity against tuberculosis induced by vaccination with major extracellular proteins of Mycobacterium tuberculosis. Proc.Natl.Acad.Sci.U.S.A. 92, 1530-1534.
26. Belisle JT et al. Role of the major antigen of mycobacterium TB in cell wall biogenesis. Science 1997;276:1420-1422

APPENDIX 1

<u>*ADVERSE EVENTS*</u>

1. Definition and Grading Intensity of Adverse Events

An adverse event is defined as any unintended change in the body structure (signs) or body function (symptoms), whether or not considered related to test product. During the entire study, subjects will be instructed to report all adverse events. All adverse events, whether volunteered, elicited or noted on physical examination, will be recorded throughout the study.

The severity of adverse events will be categorized as follows:

- MILD = Experience that is minor and does not cause significant discomfort to subject or change in activities of daily living (ADLs); subject is aware of symptoms but symptoms are easily tolerated.
- MODERATE = Experience is an inconvenience or concern to the subject and causes interference with ADLs but the subject is able to continue with ADLs.
- SEVERE = Experience significantly interferes with ADLs and the subject is incapacitated and/or unable to continue with ADLs.

2. Criteria for Determining Relationship to Test Product

The Investigator will make a determination of the relationship of the adverse event to the test product. The relationship to test product of all adverse events will be classified according to the following guidelines:

- NOT RELATED = Data available to clearly identify an alternative cause of the reaction, e.g., hemorrhage due to mechanical injury.
- UNLIKELY
  - Reasonable temporal relation to vaccination, BUT
  - Unlabeled/unexpected reaction, AND
  - The reaction can be reasonably explained by other factors (such as interventions), AND
  - Negative de-challenge, if available, OR
  - No reasonable temporal relation to vaccination.

- POSSIBLE

- Reasonable temporal relation to vaccination, AND
- Labeled/expected reaction, OR
- Unlabeled/unexpected reaction, BUT
- Other factors could have caused or contributed to the reaction (such as subject’s clinical state, concomitant therapy, and/or other interventions).

- PROBABLE
  - Reasonable temporal relation to vaccination, AND
  - Labeled/expected reaction, AND
  - The reaction cannot be reasonably explained by other factors (such as the subject’s clinical state, concomitant therapy, and/or other interventions).
- HIGHLY PROBABLE
  - Reasonable temporal relation to vaccination, AND
  - Labeled/expected reaction, AND
  - The reaction cannot be reasonably explained by other factors (such as the subject’s clinical state, concomitant therapy, and/or other interventions), AND
  - Positive de-challenge, if applicable, AND
  - Positive re-challenge, OR
  - Application/vaccination site reaction.

3 Definition of Reportable Events

The following adverse events are considered “serious reportable adverse events:”

- Death of a subject or life threatening events.
- Hospitalization (other than elective procedures or outpatient observation of <24 hour duration) or prolongation of hospitalization.
- Cancer or congenital anomaly.
- Chronic or permanent disability.
- Overdose.
- Any serious adverse event (i.e., an adverse event that is graded as serious or life-threatening in appendix 2).

APPENDIX 2

Table for Grading Severity of Adult Adverse Experiences for Vaccine Trials

Guidelines

<u>ABBREVIATIONS:</u> Abbreviations utilized in this Table include:

ULN	=Upper Limit of Normal	LLN	=Lower Limit of Normal
R_x	=Therapy	Req	=Required
Mod	=Moderate	IV	=Intravenous
ADL	=Activities of Daily Living	Dec	=Decreased

<u>ESTIMATING SEVERITY GRADE</u>

For abnormalities NOT found elsewhere in the Toxicity Table use the scale below to estimate grade of severity:

GRADE 1	Mild	Transient or mild discomfort (< 48 hours); no medical intervention/therapy required.
GRADE 2	Moderate	Mild to moderate limitation in activity – some assistance may be needed; no or minimal medical intervention/therapy required.
GRADE 3	Severe	Marked limitation in activity, some assistance usually required; medical intervention/therapy required, hospitalization possible.
GRADE 4	Life- threatening	Extreme limitation in activity, significant assistance required; significant medical intervention/therapy required, hospitalization or hospice care probable.

SERIOUS OR LIFE-THREATENING Adverse Events

ANY clinical event deemed by the clinician to be serious or life-threatening should be considered a Grade 4 Adverse Event. Clinical events considered to be serious or life-threatening include, but are not limited to: seizures, coma, tetany, diabetic ketoacidosis, disseminated intravascular coagulation, diffuse petechiae, paralysis, acute psychosis, severe depression.

<u>MISCELLANEOUS</u>

- When two values are used to define the criteria for each parameter, the lowest values will appear first.
- Parameters are generally grouped by body system.
- Some protocols may have additional protocol specific grading criteria.

APPENDIX 2 continued – Modified Table for Grading Severity of Adult Adverse Experiences for Vaccine Trials

Parameter	Grade 1<br>Mild	Grade 2<br>Moderate	Grade 3<br>Severe	Grade 4<br>Potentially<br>Life-Threatening
POTASSIUM				
Hyperkalemia	5.0 – 5.5 meq/L	5.6 – 6.0 meq/L	6.1 – 6.5 meq/L	>6.5 meq/L
Hypokalemia	3.2 – 3.4 meq/L	2.9 – 3.1 meq/L	2.5 – 2.8 meq/L	<2.5 meq/L
PHOSPHATE				
Hypophosphatemia	2.0 – 2.4 mg/dL	1.5 – 1.9 mg/dL	1.0 – 1.4 mg/dL	<1.0 mg/dL
CALCIUM – (corrected for albumin)				
Hypocalcemia	7.8 – 8.4 mg/dL	7.0 – 7.7 mg/dL	6.1 – 6.9 mg/dL	<6.1 mg/dL
Hypercalcemia	10.6 – 11.5 mg/dL	11.6 – 12.5 mg/dL	12.6 – 13.5 mg/dL	>13.5 mg/dL
MAGNESIUM				
Hypomagnesemia	1.2 – 1.4 meq/L	0.9 – 1.1 meq/L	0.6 – 0.8 meq/L	<0.6 meq/L
BILIRUBIN				
Hyperbilirubinemia	>1.0 – 1.5 x ULN	>1.5 – 2.5 x ULN	>2.5 – 5 x ULN	>5 x ULN
GLUCOSE				
Hypoglycemia	55 - 84 mg/dL	40 – 54 mg/dL	30 -39 mg/dL	<30 mg/dL
Hyperglycemia (nonfasting and no prior diabetes)	118 – 160 mg/dL	161 - 250 mg/dL	251 – 500 mg/dL	>500 mg/dL
Triglycerides	----	400 - 750 mg/dL	751 – 1200 mg/dL	>1200 mg/dL
URIC ACID				
Hyperuricemia	7.5 – 10.0 mg/dL	10.1 – 12.0 mg/dL	12.1 – 15.0 mg/dL	>15.0 mg/dL
LIVER TRANS-AMINASE (LFTs)				
AST (SGOT)	1.25 – 2.5 x ULN	>2.5 – 5.0 x ULN	>5.0 – 10.0 x ULN	> 10.0 x ULN
ALT (SGPT)	1.25 – 3.0 x ULN	>3.0 – 5.0 x ULN	>5.0 – 10.0 x ULN	> 10.0 x ULN
GGT	1.25 – 2.5 x ULN	>2.5 – 5.0 x ULN	>5.0 – 10.0 x ULN	> 10.0 x ULN
Alk Phos	1.25 – 2.5 x ULN	>2.5 – 5.0 x ULN	>5.0 – 10.0 x ULN	> 10.0 x ULN
PANCREATIC ENZYMES				
Amylase	>1.0 – 1.5 x ULN	>1.5 – 2.0 x ULN	>2.0 – 5.0 x ULN	>5.0 x ULN
Pancreatic amylase	>1.0 – 1.5 x ULN	>1.5 – 2.0 x ULN	>2.0 – 5.0 x ULN	>5.0 x ULN
Lipase	>1.0 – 1.5 x ULN	>1.5 – 2.0 x ULN	>2.0 – 5.0 x ULN	>5.0 x ULN

APPENDIX 2 continued – Modified Table for Grading Severity of Adult Adverse Experiences for Vaccine Trials

Parameter	Grade 1<br>Mild	Grade 2<br>Moderate	Grade 3<br>Severe	Grade 4<br>Potentially<br>Life-Threatening
CARDIOVASCULAR				
Cardiac Arrhythmia	------------	Asymptomatic; transient dysrhythmia, no R_x req	Recurrent/persistent dysrhythmia; symptomatic R_x req	Unstable dysrhythmia, hospitalization and R_x req
Hypertension	Transient, increase >20 mm Hg diastolic BP; no $R_x$req	Recurrent; chronic increase >20 mm Hg diastolic BP; R_x req	Acute R_x req; outpatient OR hospitalization possible	Hospitalization req OR end organ damage
Hypotension	Transient orthostatic hypotension with heart rate increased by >20 beats/min OR decreased by <10 mm Hg systolic BP, no R_x req	Symptoms OR BP decreased by <20 mm Hg systolic, correctable with oral fluid R_x	IV fluid req OR hospitalization	Mean arterial pressure <60 mm Hg, OR end organ damage, OR shock, vasopressor R_x req
Pericarditis	Minimal effuision	Mild/mod asymptomatic effusion, no R_x	Symptomatic effusion, pain, EKG changes	Tamponade OR pericardiocentesis OR surgery req
Hemorrhage, blood loss	------------	Mildly symptomatic, no R_x req	Gross blood loss OR 1-2 units transfused	Massive blood loss OR >2 units transfused
GASTROINTESTINAL				
Nausea	Mild OR transient; reasonable intake maintained	Mod discomfort OR intake decreased for <3 days	Severe discomfort OR minimal intake for $\geq$3 days	Hospitalization req
Vomiting	Mild OR transient; 2-3 episodes per day OR mild vomiting lasting <1 week	Mod OR persistent; 4-5 episodes per day; OR vomiting lasting $\geq$ week	Severe vomiting of all food/fluids in 24 hrs OR orthostatic hypotension OR IV R_x req	Hypotensive shock OR hospitalization req for IV R_x req
Diarrhea	Mild OR transient; 3-4 loose stools per day OR mild diarrhea lasting <1 week	Mod OR persistent; 5-10 loose stools per day OR diarrhea lasting $\geq$1 week	>10 loose stools/day bloody diarrhea; OR orthostatic hypotension OR electrolyte imbalance, >2 L IV fluid req	Hypotensive shock OR severe electrolyte imbalance
Oral Discomfort/ Dysphagia	Mild discomfort, no difficulty swallowing	Difficulty swallowing but able to eat and drink	Unable to swallow solids	Unable to drink fluids; IV fluids req
Constipation	------------	Moderate abdominal pain 78 hours with impaction require outpatient prescription	Requiring disimpaction or hospital treatment	Distention with vomiting OR obstipation

APPENDIX 2 – Modified Table for Grading Severity of Adult Adverse Experiences for Vaccine Trials

Parameter	Grade 1<br>Mild	Grade 2<br>Moderate	Grade 3<br>Severe	Grade 4<br>Potentially Life-Threatening
Cough (for aerosol studies)	Transient; no R_x	Treatment associated cough; inhaled bronchodilator	Uncontrolled cough; systemic R_x req	---------------
Bronchospasm Acute	Transient; no R_x; FEV1 or peak flow reduced to 70% - 80%	R_x req; normalizes with bronchodilator; FEV1 or peak flow 50% - 69%	No normalization with bronchodilator; FEV1 or peak flow 25% - 49%, retractions	Cyanosis; FEV1 or peak flow <25% OR intubated
Dyspnea	Dyspnea on exertion	Dyspnea with normal activity	Dyspnea at rest	Dyspnea requiring O_2 therapy
NEUROLOGICAL				
Neuro-cerebellar	Slight incoordination OR Dysdiadochokinesia	Intention tremor OR dysmetria OR slurred speech OR nystagmus	Ataxia requiring assistance to walk or arm incoordination interfering with ADLs	Unable to stand
Neuro-psych/mood	---------------	---------------	Severe mood changes requiring medical intervention; suicidal ideation	Acute psychosis req hospitalization ; suicidal gesture/attempt
Parasthesia (burning, tingling, etc.)	Mild discomfort; no R_x req	Mod discomfort; non-narcotic analgesia required	Severe discomfort; OR narcotic analgesia req with symptomatic improvement	Incapacitating; OR not responsive to narcotic analgesia
Neuro-motor	Mild weakness in muscle of feet but able to walk and/or mild increase or decrease in reflexes	Mod weakness in feet (unable to walk on heels and/or toes), mild weakness in hands, still able to do most hand tasks and/or loss of previously present reflex or development of hyperreflexia and/or unable to do deep knee bends to weakness	Marked distal weakness (unable to dorsiflex toes or foot drop, and mod proximal weakness e.g., in hands interfering with ADLs and/or requiring assistance to walk and/or unable to rise from chair unassisted	Confined to bed or wheel chair because of muscle weakness
Neuro-sensory	Mild impairment (decreased sensation, e.g., vibratory, pinprick, hot/cold in great toes) in focal area or symmetrical distribution	Mod impairment (mod decreased sensation, e.g., vibratory, pinprick, hot/cold to ankles) and/or joint position or mild impairment that is not symmetrical	Severe impairment (decreased or loss of sensation to knees or wrists) or loss of sensation of at least mod degree in multiple different body sites (i.e., upper and lower extremities)	Sensory loss involves limbs and trunk

APPENDIX 2 continued – Modified Table for Grading Severity of Adult Adverse Experiences for Vaccine Trials

Parameter	Grade 1<br>Mild	Grade 2<br>Moderate	Grade 3<br>Severe	Grade 4<br>Potentially<br>Life-Threatening
Arthralgia/Arthritis	Arthralgia	Arthralgia with joint effusion or moderate impairment of activity	Frank arthritis with or without effusion OR resulting in severe impairment of activity	---------------
Myalgia	Myalgia without limitation of activity	Muscle tenderness at other than injection site or with moderate impairment of activity	Frank myonecrosis OR with severe impairment of activity	---------------
SKIN				
Skin (vaccination site)	Refer to Appendix 4 for evaluation of specific changes at site of vaccination			
Skin (general)	Scattered macular or papular eruption or erythema that is asymptomatic	Scattered macular or papular eruption or erythema with pruritus or other associated symptoms	Generalized symptomatic macular, papular, or vesicular eruption	Exfoliative dermatitis or ulcerating dermatitis
URINALYSIS				
Proteinuria:<br>Random urine	1+	2 - 3+	4+	Nephrotic syndrome
Proteinuria:<br>24 hour urine	200 mg - 1 g loss/day OR <0.3% OR <3 g/l	1 – 2 g loss/day OR 0.3 – 1.0% OR 3 - 10 g/l	2 – 3.5 g loss/day OR >1.0% OR > 10 g/l	Nephrotic syndrome OR >3.5 g loss/day
Proteinuria:<br>Hematuria	Microscopic only ≤10 RBC/HPF	>10 RBC/HPF	Gross, with or without clots OR RBC casts	Obstructive OR transfusion req

APPENDIX 2 continued – Modified Table for Grading Severity of Adult Adverse Experiences for Vaccine Trials

Parameter	Grade 1<br>Mild	Grade 2<br>Moderate	Grade 3<br>Severe	Grade 4<br>Potentially<br>Life-Threatening
MISCELLANEOUS				
Fever<br>Oral>12 hours	37.7 - 38.9°C<br>(100.0 – 101.5°F)	39.0 – 39.5°C<br>(101.6 – 102.9°F)<br>OR max temp of 103°F	39.8 – 40.5°C<br>(103 - 105°F)<br>OR max temp of 103.5°F	>40.5°C (105°F)<br>OR max temp of >105°F
Headache	Mild, no R_x req, OR non-narcotic analgesia R_x	Mod; OR responds to initial narcotic R_x	Severe; intractable; OR requiring repeated narcotic R_x	Requiring hospitalization, associated with neurologic, respiratory or cardiovascular abnormalities
Allergic Reaction	Pruritus without rash at injection site	Localized urticaria at injection site	Generalized urticaria angioedema	Anaphylaxis
ADL	Normal activity reduced <48 hours	Normal activity reduced 25 - 50% >48 hours	Normal activity reduced >50%; cannot work >48 hours	Unable to care for self
Eye		Mild pain, visual changes, conjunctival erythema, abnormal slit lamp	Loss of vision, clinically diagnosed uveitis, mod-severe pain, glaucoma	--------------
